# The Maternal *ITPK1* Gene Polymorphism Is Associated with Neural Tube Defects in a High-Risk Chinese Population

**DOI:** 10.1371/journal.pone.0086145

**Published:** 2014-01-20

**Authors:** Zhen Guan, Jianhua Wang, Jin Guo, Fang Wang, Xiuwei Wang, Guannan Li, Qiu Xie, Xu Han, Bo Niu, Ting Zhang

**Affiliations:** 1 Department of Biotechnology, Capital Institute of Pediatrics, Beijing, China; 2 Beijing Municipal Key Laboratory of Child Development and Nutriomics, Capital Institute of Pediatrics, Beijing, China; National Institute of Environmental Health Sciences, United States of America

## Abstract

**Background:**

Epidemiological surveys and animal studies have revealed that inositol metabolism is associated with NTDs, but the mechanisms are not clear. Inositol 1,3,4-trisphosphate 5/6-kinase (ITPK1) is a pivotal regulatory enzyme in inositol metabolic pathway. The objective was to assess the potential impact of the maternal *ITPK1* genotypes on the inositol parameter and on the NTD risk in a NTD high-risk area in China.

**Methodology/Results:**

A case-control study of pregnant women affected with NTDs (n = 200) and controls (n = 320) was carried out. 13 tag SNPs of *ITPK1* were selected and genotyped by the Sequenom MassArray system. We found that 4 tag SNPs were statistically significant in spina bifida group (*P*<0.05). MACH was used to impute the un-genotyped SNPs in *ITPK1* locus and showed that 3 meaningful SNPs in the non-coding regions were significant. We also predicted the binding capacity of transcription factors in the positive SNPs using the bioinformatics method and found that only rs3783903 was located in the conserved sequence of activator protein-1 (AP-1). To further study the association between biochemical values and genotypes, maternal plasma inositol hexakisphosphate (IP_6_) levels were also assessed using LC-MS. The maternal plasma IP_6_ concentrations in the spina bifida subgroup were 7.1% lower than control (136.67 vs. 147.05 ng mL^−1^, *P*<0.05), and significantly lower in rs3783903 GG genotype than others (*P*<0.05). EMSA showed a different allelic binding capacity of AP-1 in rs3783903, which was affected by an A→G exchange. The RT-PCR suggested the *ITPK1* expression was decreased significantly in mutant-type of rs3783903 compared with wild-type in the 60 healthy pregnancies (*P*<0.05).

**Conclusions/Significance:**

These results suggested that the maternal rs3783903 of *ITPK1* might be associated with spina bifida, and the allele G of rs3783903 might affect the binding of AP-1 and the decrease of maternal plasma IP_6_ concentration in this Chinese population.

## Introduction

Neural tube defects (NTDs) refer to a group of serious defects and its etiology is multifactorial, involving the genetic and environmental factors [1]. As a maternal nutrition factor, inositol plays a vital role in the development of neural tube. An epidemiological study conducted by Groenen et al showed that serum levels of inositol in NTDs-affected pregnant women were significantly lower than that of healthy pregnant women and pregnant women with lower serum levels of inositol are more susceptible to NTDs by 2.6 fold [2]. In animal studies, inositol can reduce the incidence of spina bifida in the genetic model of folate-resistant NTDs [3]. The study from E. Albert Reece et al showed that supplementation of inosotol (0.08 mg/d) to pregnant mice reduced the incidence of NTDs to the next generations from 20.4% to 9.5% [4]. Recently, myo-inositol soft gelatin capsules are considered as the preventive treatment of NTDs in folate-resistant subjects [5]. In a word, results suggest that as a nutritional substance, inositol is closely related to the development of NTDs.

ITPK1 is a pivotal regulatory enzyme in the synthesis of inositol tetraphosphate (IP_4_), inositol pentakisphosphate (IP_5_), inositol hexakisphosphate (IP_6_). ITPK1 catalyzes the inositol 1,3,4-trisphosphate[I(1,3,4)P_3_] into the IP_4_ which is further transformed to IP_5_ by a 5-kinase and to IP_6_ by a 2-kinase[6]–[8]. These products regulate various cellular processes. IP_4_ inhibits the permeability of CLC3 channel and participates in the cellular signal transduction pathway; while IP_6_ is an intracellular signaling molecule, participating in the regulation of ion channels, exocytosis, endocytosis, transcription, DNA synthesis and RNA transport. Silencing or over-expression of *ITPK1* gene affected the levels of its downstream products, IP_4_, IP_5_ and IP_6_, resulting in inositol metabolic disorder [9]. The animal experiments demonstrated that *ITPK1* was expressed in the highest level in brain and the mice with reduced levels of ITPK1 developed NTDs [10]. The structure and function changes of *ITPK1* induced by polymorphisms might affect the metabolism of salt, fluid and endocrine in cell which lead to the abnormality cell proliferation, differentiation and apoptosis, therefore disrupting the normal development of neural tube. In conclusion, *ITPK1* gene might be closely correlated with NTDs.

Previous studies indicated that maternal inositol level played an important role in NTD susceptibility. These researches were focused on animal and cell models and the population studies about the *ITPK1* gene were still lack. Weather the different susceptibility is caused by the polymorphism of the key gene *ITPK1* has not been reported. For this reason, we genotyped the polymorphism of *ITPK1* gene and tested maternal plasma IP_6_ level to assess the potential impact of the maternal *ITPK1* genotypes on the inositol parameter and on the NTD risk in the high-risk area of Lvliang, Shanxi Province in China.

## Materials and Methods

### Ethics Statement

The study protocol was reviewed and approved by the Ethics Committees Review Board of Capital Institute of Pediatrics, Beijing, China. We obtained written informed consent from all parents.

### Study Population

We conducted a hospital-based case–control study from 2004 to 2009 in Shanxi Province in Northern China which have an NTD prevalence of 199.38 per 10,000 births [11]. Effective sample sizes (320 controls VS 200 cases) for case-control study were calculated by Quanto 1.1 ver. software and the power was set at 80% [12]. We chose 200 pregnant women with NTDs diagnosed with B-ultrasound examination. The pathological diagnosis of NTDs was completed by experienced pathologists according to the International Classification of Diseases, Tenth Revision, codes Q00 anencephaly, Q05 spina bifida, and Q01 encephalocele (http://apps.who.int/classifications). As for control group, women who had a live-born infant with no identified structural malformation after 1-year follow-up and who aborted for nonmedical reasons were enrolled from this region. The nonmedical aborted fetus also underwent the pathological anatomy. Any fetuses displaying pathological malformations or intrauterine growth retardation were excluded from the control group. When pregnant women were recruited, their clinical information was collected and recorded. Generally, 2 ml of venous blood was collected and stored at −20°C in local hospitals before shipping, on ice, to the study laboratories. Samples were not thawed until analysis. The population of our study was extragenetic and specifically selected and we have avoided the false positive results caused by population stratification. The collection of the samples was strictly controlled. We only had collected “anencephaly”, “spina bifida” and “encephalocele”, and there were not any other defects.

To validate the mRNA levels of the different SNPs, we recruited 60 healthy pregnant women (22–24 gestational weeks) from the Beijing Obstetrics and Gynecology Hospital, and collected the 2.5 ml venous blood in a PAXgene Blood RNA Tube (BRT). Immediately after blood collection, the PAXgene® Blood RNA Tube was gently inverted 8–10 times and stored the PAXgene® Blood RNA Tube upright at room temperature (18°C to 25°C) for a minimum of 2 hours. After that, the tube was then transferred to freezer (−20°C) before shipping, on ice, to the study laboratories.

### DNA Extraction

Genomic DNA was extracted from frozen blood samples using the Blood DNA Kit (Qiagen, Germany) according to the manufacturer’s instructions and was subsequently used for genotyping. DNA concentration and purity were determined by absorbance at 260 nm and 280 nm.

### RNA Extraction

Genomic RNA was extracted from frozen blood samples using PAXgene Blood RNA Kit (Qiagen, Germany) according to the manufacturer’s protocol and was subsequently used for RT-PCR. RNA concentration and purity were determined by absorbance at 260 nm and 280 nm.

### Single-Nucleotide Polymorphism Selection

In our study, tag SNPs were selected from the Haploview software 4.2 (Mark Daly’s lab of Broad Institute, Cambridge, MA, Britain) [13] using HapMap Genome Browser release#24 (Phase 1 & 2 - full dataset ) data [14] based on the CHB+JPT population and dbSNPs (http://www.ncbi.nlm.nih.gov/projects/SNP/) databases by the criterion that minor allele frequencies >0.2, pairwise correlation *r^2^*>0.8, giving first priority to those in coding exons, and then those underlying regulatory elements. Combined with the principles of Sequenom genotyping primer design, 13 tag SNPs were finally selected.

### Genotyping Approach

All cases and controls were genotyped for the selected SNPs of the *ITPK1* gene were using Sequenom MassARRAY System with matrix-assisted laser desorption/ionization time-of-flight (MALDI-TOF) mass spectrometry (Sequenom, San Diego, California). Two primers for Polymerase chain reaction (PCR) amplification and one primer for extension primers were designed using RealSNP (https://www.mysequenom.com/). Approximately 20 ng of genomic DNA was used to genotype each sample. The data were analyzed using Type Analysis 4.0. We had done the validation study about the genotyping accuracy in *ITPK1* gene. The Sequenom genotyping platform is medium-throughput. In order to ensure the genotyping consistency, 10% of samples were re-genotyped, and another 10% of samples were sequenced directly to validate the accuracy of the genotyping. Sequencing results were exported to Mutation Surveyor Version 3.25 (Softgenetics, State College, PA, USA; http://www.softgenetics.com) for alignment and multiple comparisons. Genotyping quality control study contained 1 blank sample, 2 repeated samples, and a >90% of genotyping call rate.

### Prediction Transcription Factor and Function Tests

We predicted the binding capacity to potential transcription factor for the different genotypes in the four positive tag SNPs using the bioinformatics method (http://www.gene-regulation.com/pub/programs/alibaba2/index.html) and found the AA genotype of rs3783903 was located in the conserved sequence of approximate transcription factors (AP-1) and there were not any other predicted transcription factor binding sites in the oligo of rs3783903. The other 3 sites were also analyzed and the results showed that they were located in the non-conserved sequences of the transcription factor binding sites. Then we applied EMSA to detect whether the rs3783903 polymorphism could change the binding ability of the sequence to the transcription factor AP-1.

### Determination of IP_6_ in Maternal Plasma

The plasma concentration of inositol (IP_6_) was detected according to the protocol of the previous study [15]. The 200 ul plasmas were transferred into the EP tubes; 20 ul of 0.1M EDTA and 20 ul of 6.1N trichloroacetic acid were added into the tubes. Then the mixture was vortexed and centrifuged at 5200 g for 10 min at 4°C. The supernatant (60 ul) was collected and added the 60 ul of 50 mM TEAA buffer, pH9. Mixtures were vortexed and taken the 50 ul sample was injected into the LC-MS apparatus (system 1). We selected 60 subjects from spina bifida groups and controls, respectively.

### Nuclear Extracts and Electromobility Shift Assay (EMSA)

Nuclear Extracts from Hela cells were prepared following the instructions and the protocol (NE-PER Nuclear and Cytoplasmic Extraction Reagents). To determine the crucial role of the rs3783903 at the region of the *ITPK1* intron, the oligonucleotides containing the nucleotide A or G at rs3783903 was designed. The AP-1 core sequence was (5′-AGTCA-3′). The sequences for these double-stranded oligonucleotides were: the probes biotinylated at the 5′ end (Sangon Biotech (Shanghai) Co, Ltd ), A (5′-tgaaagtgcAgtcaagatggtag-3′) and G (5′-tgaaagtgcGgtcaagatggtag-3′). The oligonucleotides were incubated for 20 min with an equal amount of nuclear extracts in the presence of 10% glycerol, 1 ul dIdC, binding buffer. A 200-fold excess of A or G cold probes non biotinylated at the 5′ end were used in competiton assays. The DNA-protein complexes were run on a 6% acrylamide gel and transferred to a N+ nylon membrane, cross-linked and revealed by chemiluminescence (Light Shift Chemiluminescent EMSA kit, Pierce). We used protein-specific supershift antibodies, anti-c-jun (H-79, sc-1694) and anti-c-fos (sc-52) antibodies (Santa Cruz CA, USA), to identify the components of the AP-1 transcription factor in the DNA–protein complex. These antibodies were incubated with the nuclear extracts for 30 min at room temperature before incubation with those probes. Besides, the unlabeled mutant probe was added to the wild-type labeled probe lane.

### Validation of the Differentially Expressed Genotypes by Quantitative Real-time PCR (RT-PCR)

Total RNA was extracted from the peripheral blood of the healthy women using QIAamp® RNA Blood Mini Handbook Kit(QIAamp®). Real-time qPCR analysis was performed at CWBio.Co. Ltd. (Beijing, China). Briefly, the NEB E. coli poly (A) polymerase was firstly used to add poly (A) tail to total RNA. Complementary DNA was synthesized by using the SuperRT Two Step RT-PCR Kit reverse transcriptase (CWbio.Co, Ltd) and mRNA specific reverse transcription primers. SYBR Green (UltraSYBR Mixture, CWbio.Co Ltd) uptake in double-stranded DNA was measured using ABI 7500 Sequence Detection System in the presence of SYBR-Green. We calculated *ITPK1* (Forward: ACCGCTCCAAGTCCTATGAG Reverse: GGGAAAGTCAAGCCGTTCTTC) and used this statistic to determine relative gene expression. Glyceraldehyde-3-phosphate dehydrogenase (GAPDH) was used as an internal control gene (Forward: ATGGGGAAGGTGAAGGTCG; Reverse: GGGGTCATTGATGGCAACAATA).

### Statistical Analysis

The genotype frequency of SNP was tested for Hardy-Weinberg equilibrium (HWE) in cases and controls by the Chi-square test. The indicator of linkage disequilibrium (LD), r^2^ (square of correlation coefficient), and the haplotype frequencies were analyzed using online SNPstats software at http://bioinfo.iconcologia.net/SNPstats [16]. General characteristics of the participants were presented as the mean and standard deviations (SDs) for continuous measures, while frequencies and percentages were used for categorical measures. Association of genetic polymorphisms of *ITPK1* gene with NTD risk was analyzed using univariate analysis through Chi-square test and Fisher’s exact test. The correlation between each SNP and NTDs risk was estimated by logistic regression analysis with adjustment for other variables. MACH [17], [18] was used to impute the un-genotyped SNPs in *ITPK1* locus, using the reference panel ASN data (1000 Genomes Integrated Phase 1). Association test was performed by using logistic regression. The statistical analysis was conducted using SPSS statistical analysis software, version 17.0 (SPSS, Chicago, IL, USA). Association was expressed as odds ratios (*OR*) with 95% confidence intervals (*CI*). The association was considered to be significant when the *P*-value was<0.05.

## Results

### General Characteristics of the Participants

We recruited 200 cases and 320 matched controls and conducted a hospital-based case–control study in the Lvliang mountain area of Shanxi Province in northern China and preliminarily investigated the association between *ITPK1* gene and NTDs. There were 101 anencephaly cases, 80 spina bifida cases and 19 encephalocele cases. The average genotyping call rate was >90% in all samples. Re-genotyping results showed a 100% concordance. The general characteristics of the participants are shown in Table 1. There was no significant difference between groups in maternal age, mother’s educational level, gravidity, parity and periconceptional folic acid use. The gestational week of controls was lower than that of cases.

**Table 1:** The general characteristics of the study subjects.

Characteristics	Case (N = 200^a^, %^b^)	Control (N = 320^a^, %)	*P* c
Age (year)	194	325	
<20	14 (7.2)	25 (7.7)	0.112
20–29	147 (75.8)	220 (67.7)	
>29	33 (17)	80 (24.6)	
Educational level	190	301	
< Middle school graduation	15 (7.9)	35 (11.6)	0.199
Middle school graduation	142 (74.7)	203 (67.4)	
> Middle school graduation	33 (17.4)	63 (20.9)	
Gravidity (n)	187	302	
1	13 (7)	31 (10.3)	0.45
2	20 (10.7)	33(10.9)	
≥3	154 (82.3)	238 (78.8)	
Parity (n)	186	299	
0	23 (10.22)	30 (10.04)	0.316
1	13 (7.30)	32 (10.78)	
≥2	150 (82.48)	237 (79.18)	
Periconceptional folic acid use	166	280	
No d	156 (93.9)	271 (96.8)	0.156
Yes	10 (6.1)	9 (3.2)	
Gestational week	195	304	
<21	108 (55.4)	200 (65.8)	0.041
21–29	58 (29.7)	76 (25)	
≥30	29 (14.9)	28 (9.2)	

^a^ Referes to the number of subject.

^b^ Percentages may not equal 100 because of rounding.

^c^ Chi-square test was used to calculate the p values.

^d^ The “periconceptional” refer to the month before conception and the first 3 months after conception.

### Association of Genetic Polymorphisms of *ITPK1* Gene with NTDs Risk

**Figure 1:**
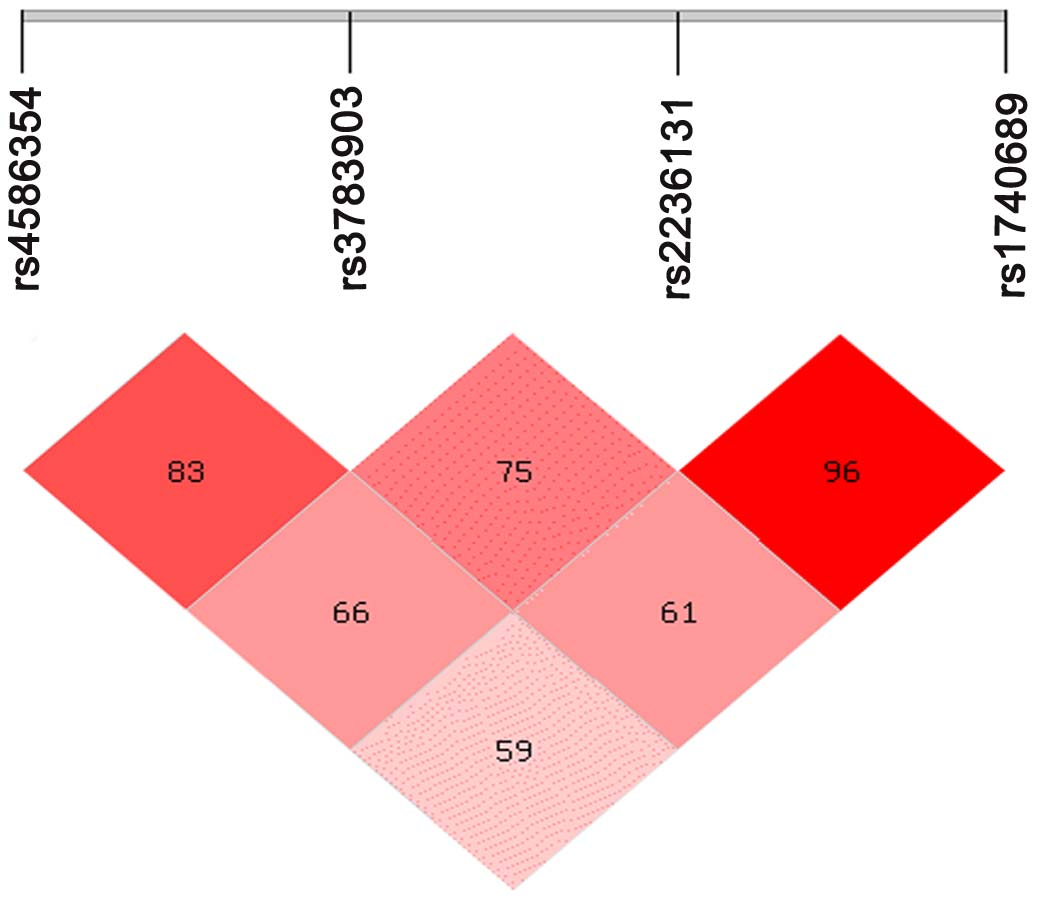
LD patterns of SNPs rs4586354, rs3783903, rs2236131 and rs1740689. The linkage patterns between the statistical difference four SNPs in all samples. The numbers in diamond represent the 100×D′ in the form of standard color scheme. The upper bar denotes the relative distance among the SNPs. The bright red box denotes D′ = 1 and LOD≥2; the pink box denotes D′<1 and LOD≥2.

The 13 tag SNPs of *ITPK1* gene were genotyped and all polymorphisms were in Hardy-Weinberg equilibrium. The study showed that the four tag SNPs were significantly different between cases and controls (Table S1). While anencepahly and spina bifida might be considered as primary neurulation defects, and encephaloceles as post-neurulation defects, we removed the encephaloceles from the analysis. Meanwhile, anencepahly and spina bifida were separately analyzed in subtypes. We re-analyzed the cases and controls and found that the statistical differences were more significant. Four SNPs (rs4586354, rs3783903, rs2236131, and rs1740689) had a high odds ratio index for NTDs (Table 2). MACH was used to impute the un-genotyped SNPs in *ITPK1* locus, using the reference panel ASN data (1000 Genomes Integrated Phase 1). Association test was performed by using logistic regression. Results showed that 64 SNPs were statistically significant (*P-value* from 5E-02 to 3.25E-08) and were in the non-coding areas, among which rs882023, rs10132322 and rs8013870 were located in the meaningful regions of the intron (Table 3). Four tag SNPs of *ITPK1* gene were analyzed, and showed that a LD was observed between all loci in this study population using http://analysis2.bio-x.cn/myAnalysis.php. Compared with the wild-haplotype CGAG, the TAGA haplotype had a significantly higher risk of NTDs (*OR* = 1.64, 95%*CI* [1.15–2.33], *P* = 0.006) (Table 4, Figure 1).

**Table 2:** The positive tag SNPs genotypes and allele frequencies in NTDs and controls.

SNP	Genotype/Allele	Cases (%)	Controls (%)	*P* a	*OR*	*OR(95%CI)*
rs4586354	TT	81 (47.4)	164 (54.1)		1	
	CT	70 (40.9)	123 (40.6)	0.48	1.15	[0.77,1.71]
	CC	20 (11.7)	16 (5.3)	0.009	2.53	[1.25,5.10]
	CT+CC	90 (52.6)	139 (45.9)	0.15	1.31	[0.90,1.91]
	T	232 (67.8)	451 (74.4)		1	
	C	110 (32.2)	155 (25.6)	0.03	1.38	[1.03,1.85]
rs3783903	AA	74 (40.9)	165 (52.2)		1	
	AG	87 (48.1)	136 (43)	0.07	1.43	[0.97,2.09]
	GG	20 (11)	15 (4.7)	0.002	2.97	[1.44,6.13]
	AG+GG	107 (57.5)	151 (47.8)	0.015	1.58	[1.09,2.28]
	A	235 (64.9)	466 (73.7)		1	
	G	127 (35.1)	166 (26.3)	0.003	1.52	[1.10,2.00]
rs2236131	GG	78 (44.3)	181 (58)		1	
	AG	79 (44.9)	115 (36.9)	0.019	1.59	[1.08,2.35]
	AA	19 (10.8)	16 (5.1)	0.004	2.76	[1.35,5.64]
	AG+AA	98 (55.7)	138 (42.3)	0.008	1.65	[1.13,2.4]
	G	235 (66.8)	477 (76.4)		1	
	A	117 (33.2)	147 (23.6)	0.001	1.62	[1.20,2.15]
rs1740689	AA	55 (31.6)	139 (44.3)		1	
	AG	88 (50.6)	143 (45.5)	0.043	1.55	[1.03,2.30]
	GG	31 (17.8)	32 (10.2)	0.002	2.45	[1.36,4.40]
	AG+GG	119 (68.4)	175 (55.8)	0.006	1.7	[1.16,2.54]
	A	198 (56.9)	421 (67)		1	
	G	150 (43.1)	207 (33)	0.002	1.54	[1.17, 2.02]

Abbreviations: NTD, neural tube defect; SNP, single-nucleotide polymorphism; *OR*, odds ratio; *CI*, confidence interval.

^a^ The *P* value remained significant after Bonferroni correction at 0.05 levels.

**Table 3:** The results of imputation and association test for the SNPs in *ITPK1* locus.

SNP	Position	Allele	MAF in Cases	MAF in Controls	*OR*	Standard Error	*P*-value	Type
rs882023	93532014	T/C	0.429	0.3258	1.82	0.16	1.59E-04	int us2k^a^
rs10132322	93487943	G/A	0.3657	0.2776	1.93	0.18	2.52E-04	int trp^b^
rs8013870	93518403	A/G	0.4311	0.3701	1.35	0.15	3.86E-02	int trp
rs55948122	93484627	T/C	0.312	0.2045	3.48	0.23	3.25E-08	int
rs3783902	93482450	A/G	0.3323	0.2209	3.26	0.22	4.37E-08	int
rs56857003	93482868	G/C	0.3346	0.2239	3.26	0.23	4.70E-08	int
rs3783904	93482298	G/A	0.3325	0.2255	3.29	0.23	7.00E-08	int
rs12433984	93482709	G/A	0.3369	0.2277	3.19	0.22	7.36E-08	int
rs3783905	93482211	C/T	0.3304	0.2239	3.26	0.23	8.22E-08	int
rs55919031	93452687	C/T	0.3068	0.2184	2.71	0.23	6.43E-06	int
rs7154944	93450468	T/G	0.3105	0.2233	2.65	0.23	9.28E-06	int
rs4905020	93447573	A/C	0.3092	0.222	2.63	0.22	1.00E-05	int
rs1815226	93439195	C/A	0.3074	0.2197	2.58	0.22	1.18E-05	int
rs3783915	93446992	C/T	0.3098	0.2234	2.58	0.22	1.36E-05	int
rs3783916	93441153	C/T	0.3098	0.2234	2.56	0.22	1.47E-05	int
rs55652648	93445141	G/A	0.3099	0.2237	2.56	0.22	1.49E-05	int
rs3783918	93439911	A/G	0.3095	0.2231	2.55	0.22	1.55E-05	int
rs4905016	93438765	C/G	0.309	0.2226	2.47	0.22	2.15E-05	int
rs12589194	93438410	T/C	0.3073	0.22	2.44	0.21	2.23E-05	int
rs3783919	93437614	A/G	0.3071	0.2199	2.43	0.21	2.30E-05	int
rs3825684	93437581	C/G	0.3073	0.2197	2.42	0.21	2.32E-05	int
rs67182772	93437363	T/C	0.3068	0.2195	2.41	0.21	2.49E-05	int
rs61446566	93436493	C/T	0.3064	0.219	2.4	0.21	2.56E-05	int
rs58809802	93550093	A/G	0.2913	0.2047	2.4	0.21	2.74E-05	int
rs57854715	93432730	T/A	0.3021	0.2137	2.34	0.21	2.98E-05	int
rs12587187	93435452	A/G	0.3063	0.2193	2.36	0.21	3.11E-05	int
rs4905017	93438821	C/A	0.3175	0.2357	2.46	0.22	3.62E-05	int
rs72704295	93430316	T/C	0.3015	0.2136	2.28	0.2	4.10E-05	int
rs8022484	93430003	G/A	0.3014	0.2137	2.28	0.2	4.33E-05	int
rs2295392	93429060	A/G	0.3023	0.2141	2.23	0.2	5.24E-05	int
rs58887679	93528433	T/C	0.3228	0.2168	1.88	0.16	8.13E-05	int
rs56159253	93528802	G/T	0.3221	0.2167	1.87	0.16	8.99E-05	int
rs1612612	93497111	A/G	0.3457	0.2487	1.96	0.17	9.91E-05	int
rs55901898	93529066	A/G	0.3219	0.2172	1.86	0.16	1.07E-04	int
rs10144329	93487652	C/T	0.3825	0.288	1.96	0.18	1.24E-04	int
rs1740598	93499081	T/C	0.3553	0.2585	1.92	0.17	1.33E-04	int
rs10144603	93487894	C/T	0.383	0.2902	1.97	0.18	1.34E-04	int
rs56041593	93544162	C/T	0.3154	0.227	2.02	0.19	1.49E-04	int
rs12587868	93494030	A/G	0.3731	0.278	1.9	0.17	1.64E-04	int
rs117634531	93525019	T/C	0.3905	0.3006	1.92	0.18	2.27E-04	int
rs10147739	93490742	T/C	0.383	0.2882	1.86	0.17	2.28E-04	int
rs55663734	93510695	G/C	0.3787	0.2883	1.8	0.17	4.49E-04	int
rs1740693	93510644	G/C	0.3785	0.2878	1.78	0.17	4.88E-04	int
rs1740694	93510531	A/G	0.382	0.2918	1.76	0.17	5.77E-04	int
rs1740594	93512947	C/G	0.3963	0.3055	1.75	0.16	5.92E-04	int
rs4905027	93467174	T/G	0.4137	0.4968	0.54	0.18	6.26E-04	int
rs1740695	93498375	A/G	0.3936	0.3015	1.73	0.16	6.41E-04	int
rs1740599	93498230	C/T	0.3935	0.3014	1.72	0.16	6.54E-04	int
rs1740696	93498149	C/T	0.3933	0.3014	1.72	0.16	6.68E-04	int
rs2236131	93540906	A/G	0.3293	0.2322	1.66	0.15	7.20E-04	int
rs3783903	93482323	G/A	0.3496	0.2612	1.6	0.15	1.92E-03	int
rs12589470	93426405	A/C	0.3163	0.2402	1.69	0.17	2.26E-03	int
rs12437283	93412166	T/C	0.4074	0.4754	0.61	0.18	5.07E-03	int
rs1740689	93544174	G/A	0.4192	0.3336	1.48	0.14	5.29E-03	int
rs2749507	93528759	A/G	0.5047	0.4257	1.45	0.14	9.45E-03	int
rs72704291	93420199	G/A	0.3387	0.2668	1.49	0.15	9.58E-03	int
rs1740691	93529213	A/C	0.5033	0.4257	1.45	0.14	9.78E-03	int
rs72704292	93423970	A/G	0.3304	0.2576	1.48	0.15	9.80E-03	int
rs2749509	93528114	A/G	0.5046	0.4255	1.43	0.14	1.02E-02	int
rs57933052	93416067	T/C	0.3414	0.2714	1.48	0.16	1.13E-02	int
rs61100258	93414467	G/A	0.3497	0.281	1.49	0.16	1.16E-02	int
rs2749508	93528319	C/T	0.5056	0.429	1.42	0.14	1.26E-02	int
rs3783924	93413261	C/T	0.3464	0.2789	1.48	0.16	1.30E-02	int
rs184040115	93524881	C/T	0.4607	0.3945	1.4	0.15	2.33E-02	int

Abbreviations: *OR*, odds ratio.

^a^ Int us2k: upstream-variant-2KB sequence variant within 2KB 5′ of gene.

^b^ Int trp: triplex forming sequences.

**Table 4:** Haplotype of 4 SNPs rs4586354, rs3783903, rs2236131 and rs1740689 in *ITPK1* gene and their relative risks for NTDs.

Haplotype	rs4586354	rs3783903	rs2236131	rs1740689	Frequency	*OR(95%CI)*	*P*
Wild-Haplotype	T	A	G	A	0.5454	1	
Mut-Haplotype	C	G	A	G	0.1898	1.64(1.15,233)	0.0063

Abbreviations: NTD, neural tube defect; *OR*, odds ratio; 95% *CI,* 95% confidence interval.

It has been reported that the different polymorphisms may hold different degrees of significance for the various NTD phenotypes [19]. So we analyzed the association of the *ITPK1* polymorphisms with NTD phenotypes (anencephaly, spina bifida). The subtypes were based on those devised by Cabaret et al [20]. The research found that in the spina bifida group, individuals with the heterozygosis genotype were at a significantly increased risk for NTDs, compared with the wide type genotype (Table 5). In our study, the subtypes of spina bifida mainly include Q05.0 cervical spina bifida with hydrocephalus (3.75%), Q05.1 thoracic spina bifida with hydrocephalus (30%), Q05.2 lumbar spina bifida with hydrocephalus (43.75%), Q05.3 sacral spina bifida with hydrocephalus (3.75%), Q05.4 unspecified spina bifida with hydrocephalus (12.5%), spina bifida combined with meningomyelocele (5%) and spinal bifida occulta (1.25%). We analyzed the subtypes of anencephaly and found that both anencephaly and anencephaly combined spina bifida were not statistically significant (Table 6). However the subtypes of spina bifida were statistically significant in the Q05.1 and Q05.2 (Table 7). The number of samples in Q05.0 (n = 3); Q05.3 (n = 3); Q05.4 (n = 10); spina bifida combined with meningomyelocele (n = 4) and spinal bifida occulta (n = 1) were too less and they were not statistically analyzed.

**Table 5:** SNPs genotypes frequencies of the 4 SNPs in the 2 subtype groups.

SNP	Genotype	Controls (%)	Anencephaly (%)	Spina Bifida (%)
rs4586354	TT	164 (54.1)	52 (53.1)	29 (39.7)
	CT+CC	139 (45.9)	46 (46.9)	44 (60.3)
	*OR*(95%*CI*)	1	1.04 (0.66,1.65)	1.79 (1.06,3.01)
	*P* a		0.85	0.027
rs3783903	AA	165 (52.2)	48 (47.5)	26 (32.5)
	AG+GG	151 (47.8)	53 (52.5)	54 (67.5)
	*OR*(95%*CI*)	1	1.21 (0.77,1.89)	2.27 (1.35,3.81)
	*P* a		0.41	0.002
rs2236131	GG	181 (58)	47 (47.5)	31 (40.3)
	AG+AA	138 (42.3)	52 (52.5)	48 (58.5)
	*OR*(95%*CI*)	1	1.45 (0.92,2.28)	2.03 (1.23,3.36)
	*P* a		0.11	0.008
rs1740689	AA	139 (44.3)	34 (27.3)	21 (27.3)
	AG+GG	175 (55.8)	63 (64.9)	56 (72.3)
	*OR*(95%*CI*)	1	1.47 (0.92,2.36)	2.12 (1.22,3.67)
	*P* a		0.11	0.007

Abbreviations: SNP, single-nucleotide polymorphism; *OR*, odds ratio; *CI*, confidence interval.

^a^ *P* values were calculated by *x* ^2^ test 2×2 contingency table for genotype distribution.

**Table 6:** SNPs genotypes frequencies of the 4 SNPs in the subtype of anencephaly.

SNP	Genotype	Contol (%)	Total Anencephaly (%)	Anencephaly & SB*(%)	Only Anencephaly(%)
rs4586354	TT	164 (54.1)	52 (53.1)	33 (52.4)	12 (52.2)
	CT+CC	139 (45.9)	46 (46.9)	30 (47.6)	11 (74.8)
	*OR*(95%CI)	1	1.04 (0.66,1.65)	1.3 (0.75,2.2)	1.02 (0.46,2.5)
	*P* a		0.85	0.34	0.85
rs3783903	AA	165 (52.2)	48 (47.5)	30 (47.6)	11 (44)
	AG+GG	151 (47.8)	53 (52.5)	33 (52.4)	14 (56)
	*OR*(95%*CI*)	1	1.21 (0.77,1.89)	1.2 (0.7,2.1)	1.4 (0.6,3.5)
	*P* a		0.41	0.5	0.4
rs2236131	GG	181 (58)	47 (47.5)	33 (53.2)	10 (41.6)
	AG+AA	138 (42.3)	52 (52.5)	29 (46.8)	14 (58.4)
	*OR*(95%*CI*)	1	1.45 (0.92,2.28)	1.15 (0.67,1.9)	1.8 (0.8,4.2)
	*P* a		0.11	0.6	0.15
rs1740689	AA	139 (44.3)	34 (27.3)	20 (32.8)	8 (34.8)
	AG+GG	175 (55.8)	63 (64.9)	41 (67.2)	15 (65.2)
	*OR*(95%*CI*)	1	1.47 (0.92,2.36)	1.63 (0.9,2.9)	1.49 (0.6,3.6)
	*P* a		0.11	0.1	0.37

* Spina Bifida.

Abbreviations: SNP, single-nucleotide polymorphism; *OR*, odds ratio; *CI*, confidence interval.

a *P* values were calculated by *x* ^2^ test 2×2 contingency table for genotype distribution.

**Table 7:** SNPs genotypes frequencies of the 4 SNPs in the subtype of spina bifida.

SNP	Genotype	Controls (%)	Spina Bifida (%)	Thoracic spina bifida with hydrocephalus (%)	Lumbar spina bifida with hydrocephalus (%)
rs4586354	TT	164 (54.1)	29 (39.7)	7 (30.4)	13 (37)
	CT+CC	139 (45.9)	44 (60.3)	16 (69.6)	22 (63)
	*OR*(95%*CI*)	1	1.79 (1.06,3.01)	2.6 (1.1,6.7)	2 (0.97,4.1)
	*P* a		0.027	0.03	0.042
rs3783903	AA	165 (52.2)	26 (32.5)	10 (41.6)	10 (28.6)
	AG+GG	151 (47.8)	54 (67.5)	14 (58.4)	25 (71.4)
	*OR*(95%*CI*)	1	2.27 (1.35,3.81)	1.5 (0.6,3.5)	2.7 (1.2,5.8)
	*P* a		0.002	0.2	0.006
rs2236131	GG	181 (58)	31 (40.3)	7 (31.8)	12 (34.3)
	AG+AA	138 (42.3)	48 (58.5)	15 (68.2)	23 (65.7)
	*OR*(95%*CI*)	1	2.03 (1.23,3.36)	2.8 (1.14,7.0)	2.5 (1.26,5.2)
	*P* a		0.008	0.027	0.009
rs1740689	AA	139 (44.3)	21 (27.3)	6 (25)	9 (25.6)
	AG+GG	175 (55.8)	56 (72.3)	18 (75)	26 (74.4)
	*OR*(95%*CI*)	1	2.12 (1.22,3.67)	2.3 (0.9,6.1)	2.3 (1.0,5.1)
	*P* a		0.007	0.05	0.04

Abbreviations: SNP, single-nucleotide polymorphism; *OR*, odds ratio; *CI*, confidence interval.

a *P* values were calculated by *x* ^2^ test 2×2 contingency table for genotype distribution.

### Prediction Transcription Factor and Function Tests

In this study, we predicted the binding capacity of the different genotypes of the four tag SNPs to potential transcription factor using the bioinformatics method and found that the AA genotype of the rs3783903 was located in the conserved sequence of approximate transcription factors AP-1, and there were not any other predicted transcription factor sites in the oligo of rs3783903. The other 3 sites were located in the non-conserved sequences of the transcription factor binding sites. Then we applied EMSA to detect that whether the rs3783903 polymorphism could change the binding ability of the sequence to the transcription factor AP-1.

**Figure 2:**
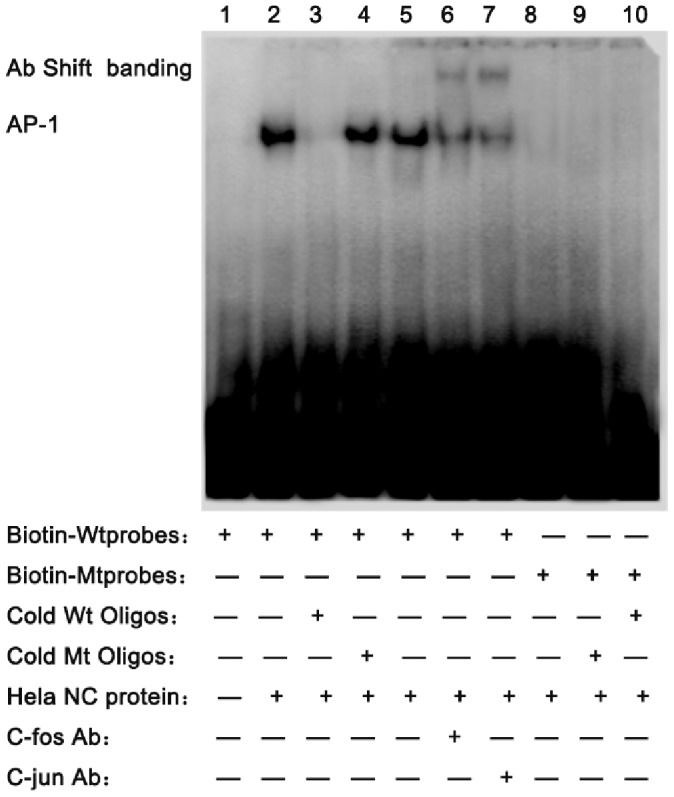
The Comparison of Wt-Oligos and Mut-Oligos in the ability of transcription factors combination. Line 1, the blank control without the nuclear extracts from HeLa cell; Line 2, the Biotin Wt-probe were incubated with the nuclear extracts from HeLa cell; Line 3–4, competition was also performed using Cold Wt Oligos and Cold Mt Oligos; Line 5, the negative control of supershift line, incubated with IgG; Line 6–7, performed with antibodies specific for c-fos, c-jun; Line 8–10, the Mut-probe were showed for the control. These results showed a different allelic binding capacity of AP-1 in rs3783903 of the *ITPK1*, which is affected by an A→G exchange.

The Wt-probe containing the AP-1 DNA-binding consensus site was incubated with the nuclear extracts from HeLa cell, shown in Figure. 2. To be certain that AP-1 is truly binding, AP-1 was incubated with an anti-c-fos and anti-c-jun antibodies with a supershift. Results showed anti-c-fos and anti-c-jun antibodies abrogated the AP-1 biding. Besides, when the unlabeled mutant probes were added to the wild-type labeled probes and the mutant probes failed to compete with the wild-type probes for AP-1 binding. Results showed a different allelic binding capacity of AP-1 in the intron region of the *ITPK1*, which was affected by an A→G exchange. Results suggested that the polymorphism affect the transcription factor binding ability. Therefore, it might regulate the expression and transcription of the *ITPK1* gene.

### The Concentration of the Maternal Plasma IP_6_

Table 8 showed that the plasma IP_6_ levels for the different genotypes between control and case were significant difference. The maternal plasma IP_6_ concentrations in the spina bifida subgroup were 7.1% lower than the control group (136.67 vs. 147.05 ngmL−1, P<0.05). Besides, compared with the other genotypes, mothers with the GG genotype had the lowest plasma IP_6_ levels, and the difference was significant (*P*<0.05).

**Table 8:** IP_6_ levels in plasma samples from maternal rs3783903 (values expressed in ng mL^−1^).

	Control (Mean±SD, n^a^)	Case (Mean±SD, n^a^)	*P* b	Total c	*P* c
AA	150.95±20.4 (20)	140.2±17.98 (20)	0.041	145.58±19.85 (40)	
AG	149.05±20.44 (20)	138.4±18.8 (20)	0.049	143.73±20.11 (40)	0.634
GG	141.15±18.91 (20)	131.4±14.79 (20)	0.033	136.28±17.4 (40)	0.01
Total	147.05±19.94(60)	136.67±17.29(60)	0.001		

a n refered to the number of subjects.

b Student’s test was used to calculate the *P* values (control VS case).

c Analysis of variance was used to calculate the *P* values and the GG genotype is significant different than other genotypes (*P* = 0.01).

### The Difference of *ITPK1* rs3783903 Expression in the 60 Healthy Women

**Figure 3:**
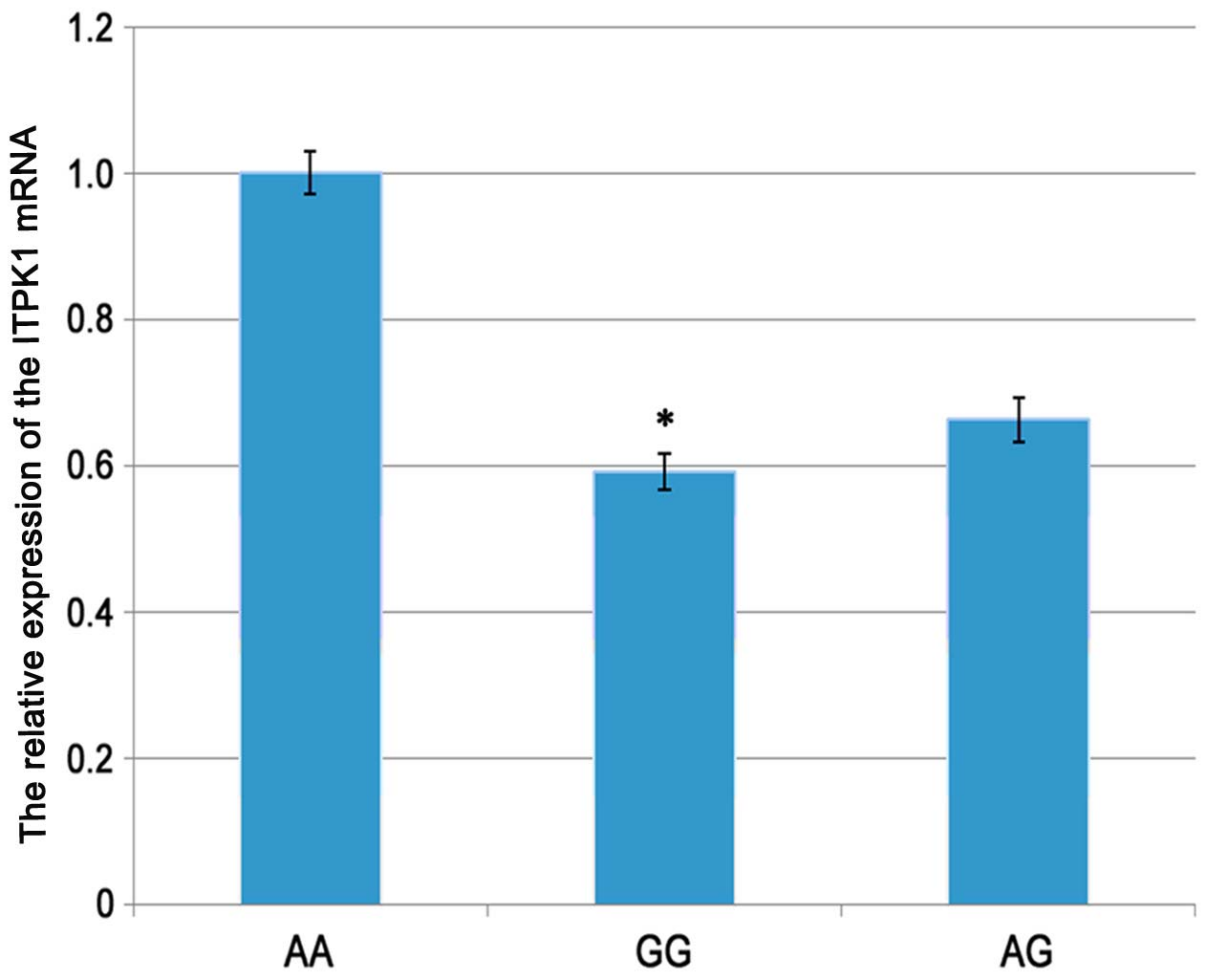
The expression of *ITPK1* gene using quantitative real-time PCR (*P<0.05). The expression of *ITPK1* was decreased significantly in mutant type of rs3783903 compared with wild type (GG: AA = 0.59; *P*<0.05).

RT-PCR was performed to examine whether different mRNA expression of rs3783903 alleles was involved in SNP-induced levels of *ITPK1* RNA (Figure. 3). The value was defined as the expression ratio of *ITPK1* to GAPDH. The expression of *ITPK1* was decreased significantly in mutant type of rs3783903 compared with wild type (GG: AA = 0.59; *P*<0.05).

## Discussion

The present study suggested that the polymorphism of *ITPK1* might be a genetic risk factor for NTDs, which provides a clue for elucidating the mechanism of NTDs induced by *ITPK1* polymorphism. The risk SNPs in *ITPK1* gene might be an indicator of the susceptibility to NTDs during the prenatal examination and serve as an individualized strategy to prevent NTDs.

As a maternal nutrition factor, inositol plays a vital role in embryo development and especially in prevention of NTDs [21]. During embryo development, inositol can be produced as L-myo-inositol-1-phosphate from D-glucose 6-phosphate [22], and it can be derived from maternal placenta which is the major source [23]. In the present study, we tested the concentration of maternal IP6 level to determine the relationship between various genotypes of rs3783903. Results showed that the plasma IP_6_ concentrations in spina bifida group were lower than that in control (*P*<0.05). IP_6_ levels in the GG sub-genotype of rs3783903 was significantly lower than that in groups of other genotypes (*P*<0.05). The *ITPK1* is a key and the rate-limiting enzyme in the synthesis of IP_4_, IP_5_ and IP_6_ in mammalian cells [24]. Deficiency of the key enzymes in the inositol metabolic pathways may decrease the inositol levels in maternal and embryonic cell, leading to the signal transduction disorder and inhibiting the cell movement, proliferation and differentiation. SNPs in the *ITPK1* dynamically regulated the metabolisms of inositol. It is supposed that the decreased maternal inositol levels might affect the placental transfer of inositol, and further affect the development of the fetus.

Our study found that there were no significant differences in general characteristics between groups except that the gestational week in controls was lower than that in cases due to the reason that some pregnant women aborted for non-medical reasons during early pregnancy. So the population we selected was representative. In the study, we found that four tag SNPs in the *ITPK1* gene in NTD-affected pregnancies were significantly associated with NTDs. In further stratified analysis we found that these SNPs were also significant in the spina bifida group (*P*<0.05, *OR*>1). Neural tube closure initiated from the tips of the neural ridges which is termed Closure 1. The failure of neural tube in Closure 1 between the midbrain and spine causes craniorachischisis. A partial failure in Closure 1 in the lumbosacral or thoracic region causes the common human birth defect, spina bifida [25]. *ITPK1* is expressed in the neural epithelium and neural crest derivatives during the developing of embryo. The SNPs, induced defects of the key enzymes (*ITPK1*) in inositol metabolic pathway might reduce IP_6_ levels in maternal and embryonic cells, resulting in disorders in the dynamic cellular behaviors in Closure 1 including cell rearrangement, cell proliferation and cell apoptosis. These events would further induce spina bifida. Different NTD phenotypes are related with different dysfunctional events and susceptibility in diverse closure sites during embryonic stages [26]. It is suggested that spina bifida is more susceptible to the polymorphism of *ITPK1* in NTD-affected pregnancies.

NTDs is a complex etiology involving both environmental and genetic factors [27]. The multi-SNPs will affect the genetically susceptibility to diseases. A difference between the haplotypes of polymorphisms can represent the combined effect of the polymorphisms or it can represent the effect of a polymorphism to which the haplotype is in LD [28]. In present study, four different tag SNPs of *ITPK1* gene (rs4586354, rs1740689, rs2236131 and rs3783903) were analyzed and we found that the haplotype TAGA increased a significant risk of NTDs (*OR* = 1.64, 95%*CI* [1.15–2.33], *P* = 0.006). The result suggested that CGAG haplotype of *ITPK1* also played a role in modulating the susceptibility to NTDs and might become a genetically susceptible index to provide the experimental evidence in screening the high risk population. The four positive tag SNPs are located in the intronic regions. MACH was used to impute the un-genotyped SNPs in *ITPK1* locus by use of the reference panel ASN data (1000 Genomes Integrated Phase 1). Results showed that the rs882023 (*P* = 1.59E-04), rs10132322 (*P* = 2.52E-04) and rs8013870 (*P* = 3.86E-02) were in the meaningful region of the intron. Polymorphisms in non-coding regions can affect the expression of a genes [29]. These SNPs may involve in the process of intron splicing and mRNA transcription and result in the aberrant expression of *ITPK1*. In addition, we predicted the binding capacity of the different genotypes of the positive tag SNPs to the potential transcription factors using the bioinformatics method. Results revealed that that AA genotype of rs3783903 was located in the conserved sequence of AP-1 with more significance than other SNPs. Many reports showed that the genetic variations in introns have impacts on modulations of gene regulation, transcript processing or chromosomal rearrangements [30], [31]. Reardon HT et al reported that six SNPs in the first intron of the *FADS2* gene were associated with the expression of *FADS1* [32]. Park TJ et al found that one common polymorphism in Intron 11, rs3816491, was most intensively associated with susceptibility to aspirin-AERD [33]. The study from Khan Sikandar G also showed that mutations in Intron 3 of the DNA repair gene affected pre-mRNA splicing in association with many skin cancers [34]. Kubota Tomoya et al found that a patient with myotonia was caused by a deletion/insertion located in Intron 21 of *SCN4A*, which is an AT-AC type II intron, and the first intronic mutation in a voltage-gatedion channel gene showing a gain-of-function defect [35]. The *ITPK1* gene contains 11 exons, 10 introns (up to 179405 bases) and the alternative splicing, suggests an indispensable role for exonic splicing enhancer sequences in intron. The rs3783903 is located in the conserved sequence of AP-1 distinguished from the other 3 sites. The rs3783903 SNP is associated with the expression levels of *ITPK1*. EMSA assays showed that the nucleo-protein complex was more effectively binded with the AP-1 of “A” allele rather than “G” allele. The rs3783903 SNP acted as a genetically modified factor and regulated alternative splicing in intron. The mRNA levels of *ITPK1* in homozygously mutated NTDs pregnancies were significantly lower than wild type in the healthy pregnant women determined by RT-PCR (*P*<0.05). The AP-1 combined with the mut-genotype might affect the promoter/enhancer activity that regulates the expression of *ITPK1* gene. Moreover, these might also regulate the splicing of *ITPK1* and influence the inositol metabolism pathway.

To our best knowledge, the present study is the first one to report a tag SNPs based selection of *ITPK1* gene polymorphisms with NTDs risk in the Lvliang mountain area of Shanxi Province in Northern China. The replication of association findings using independently collected samples is very important for the assessment of a positive finding and its generality. Besides, it is essential to provide convincing statistical evidence for association, and to rule out associations due to biases. Therefore, it is beneficial to establish the credibility of a genotype - phenotype association which is derived from candidate-gene or genome-wide association studies [36]. However, replication of our study was challenging because a suitable study would have included the same phenotype, under similar environmental background and the matched age, gravidity, parity, educational level, so a relatively larger population based cohorts would be needed to have sufficient power for replication. In our future works, we will continue to collect specimens to further evaluate data that are generated. Furthermore, our findings need to be validated in different ethnicities with a gene expression functional assay.

## Conclusions

These results suggested that the maternal rs3783903 of *ITPK1* might be associated with spina bifida, and the allele G of rs3783903 in *ITPK1* gene might affect the AP-1 binding and lead to the decrease of maternal plasma IP_6_ concentration, which might play roles in the pathogenesis of spina bifida in this Chinese population.

## Supporting Information

Table S1 The positive tag SNPs genotypes and allele frequencies in NTDs and controls. The table showed that the four tag SNPs were significantly different between cases (“anencephaly”, “spina bifida” and “encephalocele”) and controls.
(DOC)
