# Supplementary material for: The Maternal ITPK1 Gene Polymorphism Is Associated with Neural Tube Defects in a High-Risk Chinese Population
Source: PLoS One. 2014 Jan 20;9(1):e86145. doi: 10.1371/journal.pone.0086145 (PMC3896452; doi:10.1371/journal.pone.0086145)
Supplement: Table S1 — The positive tag SNPs genotypes and allele frequencies in NTDs and controls. The table showed that the four tag SNPs were significantly different between cases (“anencephaly”, “spina bifida” and “encephalocele”) and controls. (DOC) [file pone.0086145.s001.doc]

**Table S1.** The positive tag SNPs genotypes and allele frequencies in NTDs and controls.

| **SNP** | **Genotype/Allele** | **Cases (%)** | **Controls (%)** | ***P*** ^a^ | ***OR*** | ***OR******(95%CI)*** |
| --- | --- | --- | --- | --- | --- | --- |
| rs4586354 | TT | 92(48.4) | 164(54.1) |  | 1 |  |
|  | CT | 76(40) | 123(40.6) | 0.62 | 1.1 | [0.75,1.62] |
|  | CC | 22(11.6) | 16(5.3) | 0.01 | 2.45 | [1.23,4.9] |
|  | CT+CC | 98(51.6) | 139(45.9) | 0.22 | 1.26 | [0.87,1.81] |
|  | T | 260(68.4) | 451(74.4) |  | 1 |  |
|  | C | 120(31.6) | 155(25.6) | 0.04 | 1.34 | [1.01,1.78] |
| rs3783903 | AA | 85(42.5) | 165(52.2) |  | 1 |  |
|  | AG | 94(47) | 136(43) | 0.12 | 1.34 | [0.93,1.94] |
|  | GG | 21(10.5) | 15(4.7) | 0.005 | 2.72 | [1.33,5.54] |
|  | AG+GG | 115(57.5) | 151(47.8) | 0.03 | 1.48 | [1.04,2.11] |
|  | A | 264(66) | 466(73.7) |  | 1 |  |
|  | G | 136(34) | 166(26.3) | 0.008 | 1.45 | [1.1,1.89] |
| rs2236131 | GG | 91(46.7) | 181(58) |  | 1 |  |
|  | AG | 83(42.6) | 115(36.9) | 0.06 | 1.44 | [0.98,2.1] |
|  | AA | 21(10.8) | 16(5.1) | 0.006 | 2.61 | [1.30,5.24] |
|  | AG+AA | 104(53.3) | 138(42.3) | 0.03 | 1.5 | [1.05,2.14] |
|  | G | 265(67.9) | 477(76.4) |  | 1 |  |
|  | A | 125(32.1) | 147(23.6) | 0.003 | 1.53 | [1.15,2.03] |
| rs1740689 | AA | 66 (34.2) | 139(44.3) |  | 1 |  |
|  | AG | 96(49.7) | 143(45.5) | 0.08 | 1.41 | [0.96,2.09] |
|  | GG | 31(16.1) | 32(10.2) | 0.014 | 2.04 | [1.15,3.6] |
|  | AG+GG | 127(65.8) | 175(55.8) | 0.025 | 1.53 | [1.05,2.22] |
|  | A | 228(59.1) | 421(67) |  | 1 |  |
|  | G | 158(40.9) | 207（33) | 0.01 | 1.41 | [1.1, 1.89] |

Abbreviations: NTD, neural tube defect; SNP, single-nucleotide polymorphism; OR, odds ratio; CI, confidence interval.

^a^ The *P* value remained significant after Bonferroni correction at 0.05 levels.
